# Molecular diagnosis and epidemiological aspects of cutaneous Leishmaniasis in Aleppo: Current status

**DOI:** 10.1016/j.parepi.2025.e00412

**Published:** 2025-01-23

**Authors:** Lana Kourieh, Mohammad Y. Abajy, Mahasen Alkebajy, Silva Ishkhanian, Ream Nayal

**Affiliations:** a Department of Biochemistry and Microbiology, Faculty of Pharmacy, University of Aleppo, Syria; b Department of Dermatology & Venereology, Faculty of Medicine, University of Aleppo, Syria; c Department of Pharmacognosy, Faculty of Pharmacy, University of Aleppo, Syria

**Keywords:** Cutaneous leishmaniasis, kDNA PCR, ITS2 PCR, *L. major*, *L. tropica*, Aleppo boil

## Abstract

For many decades, Cutaneous leishmaniasis (CL) has been endemic in Aleppo, Syria. The situation has worsened due to the ongoing war. Currently, CL diagnosis in Syria relies mainly on conventional methods, such as clinical symptoms and microscopic examination. This study aimed to evaluate the effectiveness of PCR (polymerase chain reaction) in diagnosing suspected CL cases. Two PCR protocols were applied: kDNA PCR for CL diagnosis and ITS2 PCR to identify the *Leishmania* parasite species. The results were compared with conventional methods, and correlations between CL prevalence and demographic factors were explored.

Between January 2021 and October 2022, 105 suspected CL patients were screened at the Leishmania Center in Aleppo. kDNA PCR showed a sensitivity of 100 %, detecting positive results in 92 samples. Microscopic examination had a sensitivity of 76.1 %, identifying Leishmania amastigotes in 70 out of 92 samples. ITS2 PCR revealed that *L. tropica* was the dominant species (96.0 %) in Aleppo. Prevalence of CL was higher among men (54.3 %), the 13–25 years age group (39.1 %), and those with poor to moderate living conditions (87.0 %). Patients typically had an average of 3 lesions, with the upper extremities (55.4 %) and face (35.9 %) being the most affected areas. The study recommends using kDNA PCR for CL diagnosis due to its high sensitivity. Furthermore, the reported demographic and epidemiological data can inform public health authorities in their efforts to treat and prevent leishmaniasis in the country.

## Graphical abstract

**Figure:**
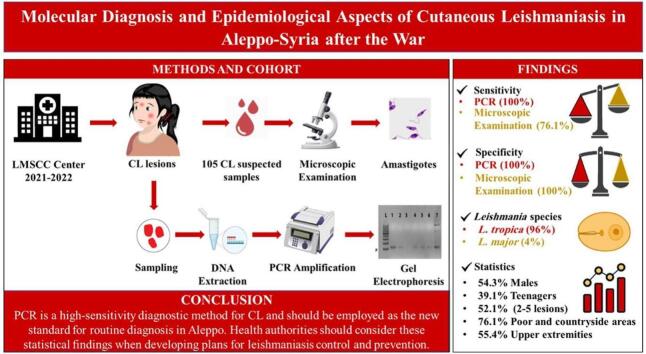

## 1 Introduction

Leishmaniasis, an endemic disease transmitted by sandflies, is caused by obligatory intracellular protozoa of the genus *Leishmania (*El Tai*, 2002**;* Akhoundi et al., *2017**)*. The disease primarily manifests in three main types: Visceral leishmaniasis (VL), also known as Kala Azar; Mucocutaneous leishmaniasis (MCL); and the most common type, Cutaneous leishmaniasis (CL). Additionally, there are clinical variants such as Post-kala-azar dermal leishmaniasis (PKDL), Diffuse cutaneous leishmaniasis (DCL), and Leishmaniasis recidivans (LR) (El Tai, 2002). Leishmaniasis poses a threat to over 350 million people across 98 countries worldwide. Each year, approximately 2 million cases are diagnosed, with 0.7–1.2 million of these being CL cases (Alvar et al., 2012; Peacock et al., 2007).

Historically, Syria has been one of the countries with the highest number of CL cases in the Middle East and North Africa (MENA) region and globally (McDowell et al., 2011). Aleppo, in particular, has been significantly affected, earning it the nickname “ALEPPO BOIL” (Douba et al., 2012). According to the World Health Organization (WHO), the number of CL cases in Syria sharply increased, with 89,357 new cases recorded across the country between January and December 2019, a substantial proportion of which occurred in Aleppo (World Health Organization, 2023). An epidemiological study conducted in the Middle East in 2022 further confirmed that Syria has the highest number of CL cases compared to Saudi Arabia, Yemen, and Iraq, based on reported cases from 2013 to 2020 (Knight et al., 2023). The recent war, population displacement, healthcare system challenges, and infrastructure disruptions have all contributed to this rise in cases.

Leishmaniasis has at least 21 pathogenic species (Akhoundi et al., 2016). In Syria, the predominant type is anthroponotic CL, caused by the species *Leishmania tropica*. However, there have also been reported cases of zoonotic CL caused by *Leishmania major (*Alvar et al., *2012**;* Rioux et al., *1990**)*. Accurate diagnosis of CL is crucial for initiating appropriate treatment, as delayed treatment can lead to serious disability and permanent scarring. Additionally, species classification is essential for epidemiological studies and treatment decisions (El Tai, 2002; Magill, 2005).

Traditionally, CL diagnoses have relied on clinical symptoms and microscopic examination (Magill, 2005; Vega-Lopez, 2003). However, these methods have limitations. Microscopic examination's sensitivity varies based on the number of amastigotes in the lesions and the examiner's expertise (Magill, 2005). Furthermore, CL symptoms often overlap with those of other skin conditions, such as eczema, bacterial skin infections, skin cancer, and skin tuberculosis (Çulha et al., 2020).

Molecular methods based on the amplification of *Leishmania* DNA through polymerase chain reaction (PCR) have become widely used for diagnosing CL (Al-Jawabreh et al., 2018). These methods offer several advantages over traditional diagnostic approaches. They address the challenge of detecting low amastigote loads (Mann et al., 2021) and allow for species-specific diagnosis (Al-Jawabreh et al., 2018; Mouttaki et al., 2014; Moreira et al., 2018).

The kDNA gene has recently been a primary target for PCR-based leishmaniasis diagnosis (Koltas et al., 2016). Additionally, the internal transcribed spacers (ITS1&2) region of the ribosomal DNA repeat unit has been extensively utilized to differentiate *Leishmania* species responsible for Old World CL (Azmi et al., 2011; De Almeida et al., 2011).

This study aims to emphasize the importance of using modern diagnostic techniques like PCR for accurate and effective CL diagnosis in one of the world's most endemic countries. Furthermore, it seeks to identify the most common *Leishmania* species causing CL and explore correlations between CL prevalence and demographic and epidemiological factors, such as gender, age, and living conditions.

## 2 Materials and methods

### 2.1 Sampling

Between January 2021 and October 2022, this study collected 105 specimens from patients suspected of CL who had experienced one or more skin lesions persisting for two weeks or longer without improvement. These patients were referred to the Leishmania, Malaria, and Schistosomiasis Control Center (LMSCC) in Aleppo city. Initially, all patients underwent clinical examination by a dermatologist. Data were then gathered through a questionnaire that included information on age, gender, living status, and previous treatment history.

To prepare the samples, lesions were cleaned and sterilized with disinfectant. A small incision was made at the sanitized edge of each lesion using a disposable surgical blade (No. 11). The skin sample was placed on a clean glass microscopic slide and stained with Wright's Giemsa for subsequent microscopic examination (Bensoussan et al., 2006).

For PCR analysis, sterile Whatman filter papers (FP) measuring 110 mm were pressed onto the lesion at the site of the incision. The sample sizes varied for each patient based on lesion size: small lesions covered approximately 20 mm of the FP, while larger lesions extended over an area ranging from 30 to 40 mm. The filter papers were then allowed to air dry completely, individually enveloped in aluminum foil, and stored at 4 °C until DNA extraction was performed.

### 2.2 DNA extraction

Each sample was taken from the filter paper using a disposable surgical blade and transferred to a 1.5 mL Eppendorf tube. DNA extraction followed the phenol-chloroform method described by Fata *et al* (Fata et al., 2009), with some adjustments. Specifically, the lysis buffer volume was increased to 400 μl (instead of 200 μl) to account for buffer loss absorbed by the filter paper. Additionally, the incubation time was extended at −20 °C to 30 min (from the original 10 min) to enhance DNA sedimentation. Finally, each sample was centrifuged at 15,000 rpm for 15 min at 4 °C (instead of 12,000 rpm) to maximize DNA yield. To validate the extraction process, DNA samples were analyzed using gel electrophoresis with 1× Tris-acetate-EDTA (TAE) buffered 1.0 % agarose gels, and the bands were visualized under UV light. As a negative control, we also tested an extracted solution from a blank filter paper to ensure no DNA contamination in the phenol-chloroform protocol. While a blood sample from a confirmed CL patient was used as a positive control.

### 2.3 PCR analysis

two PCR methods were conducted using the VIVANTIS DNA amplification Kit. The first method, kDNA PCR, aimed to diagnose CL. It amplified a 120 bp fragment in the constant region of kDNA minicircles, which is common to all *Leishmania* species. The reaction was carried out on all 105 samples using the forward and reverse primers respectively 13 A (5′-GTG GGG GAG GGG CGT TCT-3′) and 13B (5′-ATT TTC CAC CAA CCC CCA GTT-3′) basically as described by Reale *et al* (Reale et al., 1999), with slight modifications: the reaction volume was 25 μl, and 2 μl of DNA template was used instead of 10 μl to prevent DNA overload.

For species identification, ITS2 PCR was performed on kDNA PCR-positive samples by using specific forward and reverse primers respectively LeishF (5′-CAA CAC GCC GCC TCC TCT CT-3′) and LeishR (5′-AAA CAA AGG TTG TCN GGG −3′) designed by Shahbazi et al. (Shahbazi et al., 2008). These primers target the ITS2 region (AJ300485.1), which ranges from 412 to 940 bp and lies between the 5.8S rRNA and LSU rRNA genes. Amplification of the ITS2 region with these primers produces variable-length fragments due to ITS2 polymorphism, allowing species typing. The reaction was done in a final volume of 25 μl containing 2.5 μl MgCl2, 5 μl 10× Vi Buffer A, 1 μl dNTPs, 2 μl each primer, 0.5 μl of the DNA template, and 0.5 μl of DNA Taq polymerase. Cycling conditions were as follows: initial denaturation 94 °C for 5 min; followed by 30 cycles of 94 °C for 35 s, 56.8 °C for 45 s, 72 °C for 45 s and a final extension step at 72 °C for 7 min. To prevent any contamination-related bands, reaction buffers without DNA templates were included as negative controls. Additionally, DNA extracted from the cultured Syrian Strain of *Leishmania tropica* was used as a positive control in each PCR analysis—a valuable gift from the Department of Pharmacognosy, Faculty of Pharmacy, Aleppo University*.*

The amplified products were analyzed by gel electrophoresis on 2.0 % agarose gels buffered with 1× Tris-acetate-EDTA (TAE) and were visualized under UV light.

### 2.4 Statistical analysis

Data were analyzed using the Statistical Package for the Social Sciences (SPSS) software, version 16 (SPSS Inc., Chicago, IL, USA). The Chi-square test was applied to compare PCR and microscopic examination for diagnosing CL. Additionally, sensitivity, specificity, positive predictive values (PPV), negative predictive values (NPV), and Cohen's kappa coefficient (κ) were identified. Cohen's kappa coefficient assesses the agreement between two tests beyond what is expected by chance, with 0 representing chance agreement and 1 representing perfect agreement. Furthermore, the Chi-square test was used to explore potential correlations between the prevalence or frequency of CL and variables such as age, gender, living status, and lesion location. *P-values* < 0.05 were considered statistically significant.

## 3 Results

### 3.1 Verification of DNA extraction step

In this study, the traditional phenol-chloroform method was applied to extract DNA from all specimens. Subsequently, an electrophoresis was conducted to validate the effectiveness of this extraction method.

(Fig. 1).

**Fig. 1:**
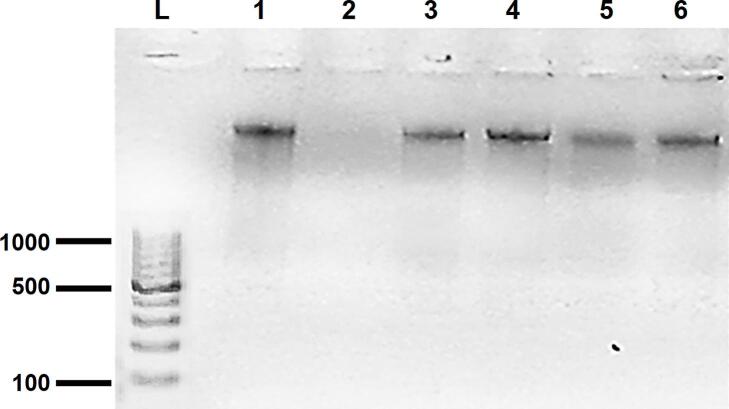
Verification of DNA extraction method: Lane L 100 bp DNA Ladder, lane 1 positive control = a blood sample obtained from a patient diagnosed with CL, lane 2 negative control = a blank filter paper, lanes 3–6 isolated DNA samples from patients suspected to have CL.

### 3.2 Evaluation of PCR as a diagnostic method

**Fig. 2:**
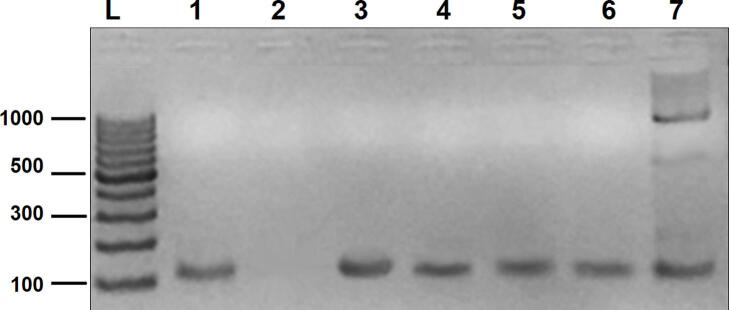
Shows kDNA PCR results analyzed by agarose gel electrophoresis: Lane L 100 bp DNA Ladder, lane 1 positive control = extracted DNA from cultured Syrian Strain of *Leishmania tropica*, lane 2 negative control = reaction buffers without DNA template, lanes 3–7 specimens from patients with CL (120 bp).

Specimens from 105 suspected CL patients were analyzed using two diagnostic methods: microscopic examination and kDNA PCR. The results obtained from each method were compared (Table 1). Among the 105 collected samples, 70 (66.6 %) tested positive based on microscopic examination of smears. The kDNA PCR correctly identified all 70 positive specimens detected by microscopic examination (Fig. 2). Additionally, 22 out of 35 cases that were negative using the traditional method were found to be positive by kDNA PCR. For the 13 samples that tested negative with both microscopic examination and kDNA PCR, they were confirmed as non-Leishmania infection status by consulting dermatologists at the LMSCC and closely monitoring their clinical conditions and lesion development. Consequently, the sensitivity of microscopic examination was 76.1 %, while kDNA PCR achieved 100 % sensitivity. In kDNA PCR, there were no false negatives, resulting in a negative predictive value (NPV) of 100 %. However, with microscope examination, 22 samples were incorrectly diagnosed as false negatives, leading to an NPV of 37.0 %. Notably, there were no false positive results with either method (specificity: 100 %, positive predictive value (PPV): 100 %). Overall, the prevalence rate of cutaneous leishmaniasis among suspected patients was 87.6 %, and the agreement level between PCR and smear diagnosis was moderate (Cohen's kappa coefficient: 0.441). Statistically, there was a significant difference between the two methods in diagnosing CL (*P* < 0.001).

**Table 1:** Comparison of kDNA PCR with the traditional microscopic examination method to diagnose cutaneous leishmaniasis.

Method	C-Pos	C-Neg	C-Pos%	C-Neg%	Sensitivity (%)	Specificity (%)	PPV (%)	NPV (%)
Microscopic examination	70	35	66.7 %	33.3 %	76.1 %	100 %	100 %	37.1 %
kDNA PCR	92	13	87.6 %	12.4 %	100 %	100 %	100 %	100 %

**Fig. 3:**
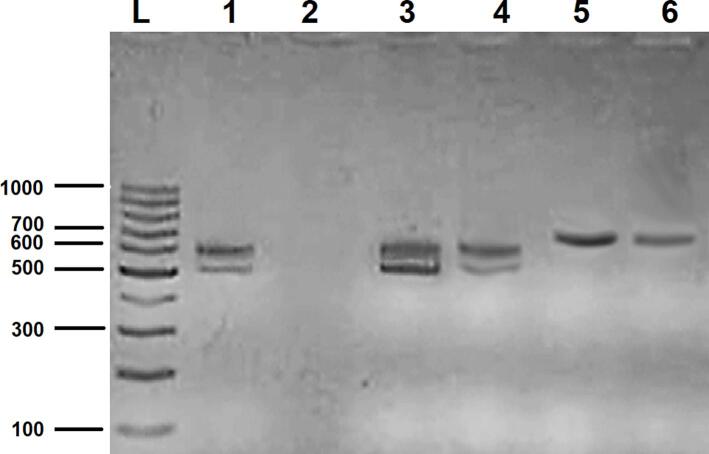
Identification of *Leishmania* species using ITS2 PCR: Lane L 100 bp DNA Ladder, lane 1 positive control = extracted DNA from cultured Syrian Strain of *Leishmania tropica*, lanes 2 negative control = reaction buffers without DNA template, lane 3–4 *L.tropica* (520 and 620 bp), lane 5–6 *L. major* (670 bp).

*Leishmania* species identification was performed using ITS2 PCR on kDNA-positive CL specimens. The sensitivity of ITS2 PCR was lower (90.9 %) compared to kDNA PCR. The results indicated that L. *tropica* was responsible for the majority of infections, while only a few cases were attributed to L. *major*. No traces of L. *infantum* were observed (Table 2, Fig. 3).

**Table 2:** Results of ITS2 PCR to determine the most common *Leishmania* species in Aleppo.

Species	%	Size estimated (bp)of ITS2 amplicons
*L. tropica*	96.0	520,620
*L. major*	4.0	670
*L. infantum*	0	603

The previous figures represent typical examples from all the analyzed images of the samples collected during this study.

### 3.3 Frequency of CL cases based on gender and age group

In this study, out of 105 patients, 92 were confirmed to have CL. Among them, 50 (54.3 %) were male, and 42 (45.7 %) were female. The highest infection rate (39.1 %) occurred in the teenage age group (13–25 years old), while the lowest rate (4.3 %) was observed in older adults aged over 50 years. Demographic and epidemiological data for all confirmed CL patients are presented in Table 3.

**Table 3:** Demographic and epidemiological characteristics of CL patients and correlation of CL prevalence to certain variables.

Characteristics	No. infected (%)	χ^2^	*P*-value
Gender		0.238	0.626
Male	50(54.3)		
Female	42(45.7)		
Age group		4.259	0.235
Infants & children (0-12y.o)	23(25.0)		
Teenagers (13-25y.o)	36(39.1)		
Adults (26-50y.o)	29(31.5)		
Older adults (>50y.o)	4(4.3)		
Living situation		0.604	0.739
Poor	40(43.5)		
Moderate	40(43.5)		
Good	12(13.0)		
Place of residence		2.302	0.512
City-poor	45(48.9)		
City-intermediate	13(14.1)		
City-rich	9(9.8)		
Countryside	25(27.2)		
Number of lesions		1.05	0.001
1	34(37.0)		
2–5	48(52.1)		
6–10	7(7.6)		
> 10	3(3.3)		
Lesion location			
Head	4(4.3)	0.588	0.44
Face	33(35.9)	6.8	0.01
Trunk	8(8.7)	1.224	0.27
Upper extremities	51(55.4)	14.01	0.001
Lower extremities	28(30.4)	5.39	0.02
Pretreatment		0.928	0.336
Yes	30(32.6)		
No	62(67.4)		
Previous infection		1.608	0.205
Yes	15(16.3)		
No	77(83.7)		
Infected relatives		6.099	0.014
Yes	31(33.7)		
No	61(66.3)		

### 3.4 Frequency of CL cases based on living status and place of residence

**Fig. 4:**
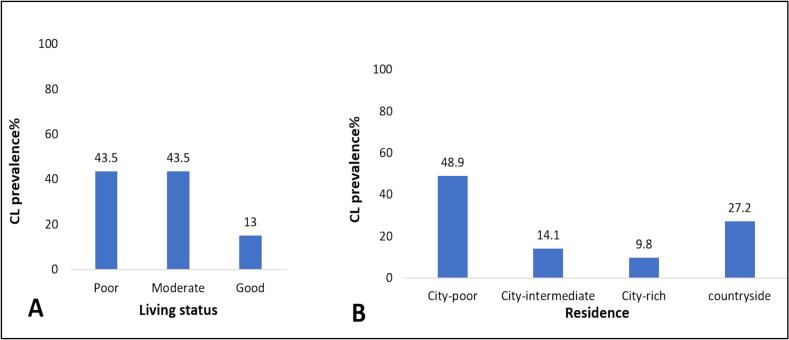
A: CL prevalence% according to living status B: CL prevalence% according to residence.

The living conditions and residence of patients played a crucial role in identifying the most affected areas in Aleppo. Recent deterioration in living conditions impacted infection rates. 87.0 % of patients experienced poor to moderate living conditions, while only 13.0 % of the patients had a good general condition. Regarding residence, the percentage of patients who lived in poor, intermediate, and affluent areas of the city were 48.9 %, 14.1 %, 9.8 %, respectively. Additionally, 27.2 % of patients hailed from rural areas (Fig. 4) (Table 3).

### 3.5 Number of lesions and their location on the body

Of 92 cases, 34(37.0 %) of patients had a single lesion, 48 (52.1 %) had 2–5 lesions, 7 (7.6 %) had 6–10 lesions, and 3 (3.3 %) had more than 10 lesions (Table 3). The number of lesions ranged between 1 and 16, The mean number of lesions per patient was 3, and there was a significant correlation between the number of lesions and CL incidence (*P* = 0.001).

Lesions were widespread across the body: locating on the upper extremities in 51 cases (55.4 %), on the face in 33 cases (35.9 %), on the lower extremities in 28 cases (30.4 %), on the trunk in 8 cases (8.7 %) and on the head in 4 cases (4.3 %). Notably, there was a significant correlation between lesion locations (upper extremities, face, and lower extremities) and CL incidence (*P* = 0.001, *P* = 0.01, *P* = 0.02, respectively) (Table 3).

### 3.6 Duration of infection, pretreatment of the lesion, prior infections, and the presence of infected family members

The duration of infection and early diagnosis of CL play a crucial role in reducing complications. Among the patients, only 2.2 % visited the LMSCC within two weeks of infection. The majority (90.2 %) sought medical attention between 1 and 6 months after infection. A smaller percentage (4.3 %) visited LMSCC after 7 to 9 months, and 3.3 % waited over a year before seeking care.

During this period, 32.6 % of patients treated their lesions under dermatologist supervision or independently, using various methods such as topical antibiotics, sterilizers, steroid ointments, cautery, and alternative therapies. In contrast, 67.4 % of patients received no treatment before diagnosis (Table 3).

Regarding medical history, only 16.3 % of patients had a previous CL infection, while the majority (83.7 %) experienced CL for the first time. Additionally, 33.7 % of patients had close-infected relatives at the same time, while 66.3 % did not have any infected relatives. Interestingly, there was a statistically significant correlation between having infected relatives concurrently and the prevalence of CL (*P* = 0.014). However, no significant correlation was found between variables such as gender, age, living status, place of residence, pretreatment, and previous CL infection in terms of prevalence rate (*P* > 0.05) (Table 3).

## 4 Discussion

In recent years, PCR has gained widespread use for diagnosing cutaneous leishmaniasis (CL). However, in Syria, the primary reliance for CL diagnosis remains on clinical symptoms and microscopic examination. Although the sensitivity of microscopic examination depends on the amastigote load in the lesions and requires an experienced examiner (Magill, 2005), it has been the standard method for diagnosing CL over the past decades. Parasite culture, while applied in neighboring countries, is not widely used in Syria due to contamination risks and time-consuming procedures (Bensoussan et al., 2006). Given the large number of patients in the country, PCR has become essential for rapid and accurate diagnosis due to its high sensitivity and specificity.

Several targets have been discovered and studied for diagnosing leishmaniasis using PCR. Notably, two studies compared different targets to determine their sensitivity. Bensoussan et al. (Bensoussan et al., 2006) evaluated kDNA PCR, ITS1 PCR, and the spliced leader mini-exon PCR. The results indicated that kDNA PCR was the most sensitive target (98.7 %), while ITS1 PCR had lower sensitivity (91.0 %). Another study by Koltas et al. (Koltas et al., 2016) also compared various targets, confirming that kDNA is the ideal genomic target for diagnosing leishmaniasis.

In this study, microscopic examination yielded 70 positive results out of 105 suspected cases, with a sensitivity value of 76.1 %. This sensitivity aligns with findings from other studies (Bensoussan et al., 2006; Shahbazi et al., 2008; Nateghi Rostami et al., 2020; Chargui et al., 2005), where sensitivity ranged from 74.4 % to 80.8 %. However, there remains a risk of at least 20.0 % false negatives, highlighting the limitations of relying solely on microscopic examination for accurate leishmaniasis diagnosis.

As anticipated, kDNA PCR outperformed the traditional method and ITS2 PCR, achieving 100 % sensitivity. Specifically, 22 samples tested positive only with kDNA PCR. This heightened sensitivity is attributed to kDNA PCR's ability to detect lesions containing very low parasite numbers, which may go undetected by microscopic examination.

In addition to diagnosing leishmaniasis, PCR was also used to identify the *Leishmania* species. This is important for investigating the most prevalent species in the city and understanding its relationship with clinical manifestations and disease epidemiology (Al-hucheimi et al., 2009), especially given the changing demographics in Aleppo due to war conditions and population displacement.

Determining the species is crucial for selecting appropriate therapy. PCR followed by restriction fragment length polymorphism analysis (RFLP) has been shown to be a suitable method for identifying *Leishmania* species, as confirmed by various studies (De Almeida et al., 2011; Kumar et al., 2007; Hijawi et al., 2019). However, RFLP is more expensive and time-consuming compared to the ITS PCR method.

In this study, ITS2 PCR was used to identify the species. Among the isolates, 96.0 % were L. *tropica*, and 4.0 % were L. *major*. These results align with other studies on Old World CL, where most cases were attributed to either L. *major* or L. *tropica* (Bensoussan et al., 2006; Shahbazi et al., 2008; Al-hucheimi et al., 2009; Bizri et al., 2021).

In Aleppo, *L. tropica* is the predominant species, as confirmed by Douba et al. (Douba et al., 2012). Similarly, Bizri et al. and El Safadi et al. (Bizri et al., 2021; El Safadi et al., 2019) in Lebanon also found that L. *tropica* is more dominant than L. *major*. This result is expected due to increased CL cases in Lebanon coming from population displacement from Syria in recent years. Conversely, *L. major* is the dominant species in Iraq (Al-hucheimi et al., 2009; Al-Warid et al., 2017) and Jordan (Hijawi et al., 2019).

In this study, the prevalence of CL was higher in males (54.3 %) than in females (45.7 %). This finding aligns with research by Al-Warid et al. and Farahmand et al. (Al-Warid et al., 2017; Farahmand et al., 2011), where the number of infected males was also greater than that of females. This difference may be attributed to factors such as clothing habits, occupational exposure, and travel to endemic regions (Moein et al., 2018).

As predicted, the highest infection rate (48.9 %) occurred in the impoverished areas of the city. Additionally, 87.0 % of the infected individuals had poor to intermediate living conditions. These findings confirm a strong association between leishmaniasis and poverty, which can be attributed to inadequate housing conditions; such as cracked walls that serve as resting places for sandflies, poor sanitation, and irregular garbage collection that creates breeding grounds for these insects (Yamey, 2002; Ranjan et al., 2005; Costa et al., 2005).

Regarding the number of lesions, the highest percentage of patients (63.0 %) exhibited more than one lesion. This finding aligns with a previous study conducted in Jordan (Hijawi et al., 2019), which confirmed that Syrian refugees who were infected with CL in Syria before their displacement to Jordan had a higher number of lesions compared to Jordanian patients. However, our results diverge from a study by Farahmand et al. in Iran (Farahmand et al., 2011), where the majority of patients had only one lesion. This discrepancy suggests that Syrians are more susceptible to sandfly bites due to inadequate control of the sandfly vector, likely exacerbated by the weakened healthcare infrastructure resulting from the country's decade-long war (Hayani et al., 2015).

In most cases (90.2 %), patients reported a history of lesions appearing 1–6 months before diagnosis. In a study by Talari et al. (Talari et al., 2006) in Iran, the average period for reviewing dermatology clinics was 8–12 months. Additionally, 32.6 % of patients had received various inappropriate treatments before their correct diagnosis. These findings highlight the lack of awareness and limited availability of information among the population in Aleppo, which contributes to delays in seeking medical attention at the LMSCC (Abazid et al., 2012). Since the appearance of CL lesions can resemble other skin conditions, some patients received treatments assuming their lesions were acne, bacterial infections, or eczema. It's crucial to address these delays, as although CL is not fatal, delayed diagnosis and treatment can increase the risk of contamination and morbidity associated with the disease (Garapati et al., 2018).

In our study, 16.3 % of patients had a history of previous CL infections. According to the Centers for Disease Control and Prevention (CDC), individuals can experience multiple CL infections throughout their lifetime (Centers for Disease Control and and Prevention, 2020). Some prior studies explored the potential for cross-immunity between different *Leishmania* species. This concept has practical applications, as vaccination with one *Leishmania* species may induce protective responses against other species (Noorpisheh Ghadimi et al., 2019; Porrozzi et al., 2004).

Furthermore, due to the country's challenging economic situation, many families have hosted displaced relatives. As a result, 33.6 % of patients had close relatives who were concurrently infected. This correlation was statistically significant in relation to the incidence of CL (*P* = 0.014).

## 5 Conclusion

In summary, the kDNA PCR is a highly sensitive diagnostic method for cutaneous leishmaniasis (CL) and should be the new standard for routine diagnosis in Aleppo. However, when identifying the *Leishmania* species causing the lesions is necessary, the ITS2 PCR is a suitable tool. Additionally, this study provides valuable demographic and epidemiological data on CL frequency, aiding public health specialists in making informed decisions about management methods for controlling CL in Aleppo.

## Ethics approval and consent to participate

All experiments were conducted according to the Code of Ethics of the World Medical Association Declaration of Helsinki. The protocols employed were approved by the ethics committee at the University of Aleppo's Faculty of Pharmacy (certificate Nr: 6/IV, 2022). Informed consent was obtained from all participating adults, and consent for including infants and children was secured from parents or guardians. Data privacy and confidentiality have been maintained.

## CRediT authorship contribution statement

**Lana Kourieh:** Writing – original draft, Resources, Project administration, Conceptualization. **Mohammad Y. Abajy:** Supervision, research design, formal analysis, review of the manuscript. **Mahasen Alkebajy:** Microscopic results review. **Silva Ishkhanian:** Formal analysis, review of the manuscript. **Ream Nayal:** statistical study, culturing of *Leishmania tropica*, manuscript review.

## Declaration of competing interest

The authors declare that they have no known competing financial interests or personal relationships that could have appeared to influence the work reported in this paper.
